# The Role of Omega-3 in Attenuating Cardiac Remodeling and Heart Failure through the Oxidative Stress and Inflammation Pathways

**DOI:** 10.3390/antiox12122067

**Published:** 2023-12-01

**Authors:** Taline Lazzarin, Danilo Martins, Raquel S. Ballarin, Marina G. Monte, Marcos F. Minicucci, Bertha F. Polegato, Leonardo Zornoff

**Affiliations:** Internal Medicine Department, Botucatu Medical School, São Paulo State University (UNESP), Botucatu 18600-000, Brazil; taline.lazzarin@unesp.br (T.L.); d.martins@unesp.br (D.M.); raquel.ballarin@unesp.br (R.S.B.); marina.monte@unesp.br (M.G.M.); marcos.minicucci@unesp.br (M.F.M.); bertha.polegato@unesp.br (B.F.P.)

**Keywords:** oxidative stress, cardiac failure, remodeling, fatty acids, omega-3

## Abstract

Cardiac remodeling is defined as molecular, cellular, and interstitial changes that manifest clinically as alterations in the size, shape, and function of the heart. Despite the pharmacological approaches, cardiac remodeling-related mortality rates remain high. Therefore, other therapeutic options are being increasingly studied. This review highlights the role of omega-3 as an adjunctive therapy to attenuate cardiac remodeling, with an emphasis on its antioxidant and anti-inflammatory actions.

## 1. Introduction

Cardiac remodeling is defined as molecular, cellular, and interstitial changes that manifest clinically as alterations in the size, shape, and function of the heart. Cardiac dysfunction is the main consequence of cardiac remodeling; therefore, it plays a critical role in the onset and progression of ventricular dysfunction [1,2,3].

Damage to cardiomyocytes owing to several conditions, including myocardial infarction (MI), myocarditis, hypertension, diabetes, valvular diseases, toxicity, inflammation, arrhythmia, and genetic diseases, can lead to cardiac remodeling [1,2].

The clinical diagnosis of remodeling is based on the detection of morphological changes, which can be evaluated by image methods like echocardiography, ventriculography, and cardiac nuclear magnetic resonance [1].

Although the elucidation of all pathophysiological mechanisms in cardiac remodeling is lacking, some of those implicated in this condition are cellular death, inflammation, fibrosis, oxidative stress, neurohumoral activation, cardiomyocyte energy metabolism deficit, and alterations in contractile proteins and calcium channels. Some pharmacological treatments that act on these targets include beta-blockers, angiotensin-converting enzyme inhibitors, aldosterone antagonists, and SGLT-2 inhibitors. Despite these pharmacological approaches, cardiac remodeling-related mortality rates remain high; therefore, other therapeutic options are being increasingly studied [1,3,4]. Considering this background, we highlight the body of clinical evidence available to support the use of omega-3 polyunsaturated fatty acids (ω3-PUFAs) as an adjunctive therapy to attenuate cardiac remodeling.

Polyunsaturated fatty acids are lipid components comprising a hydrocarbon chain terminating with a carboxylic acid group (–COOH) at the polar hydrophilic end and a nonpolar hydrophobic methyl group (–CH3) at the other end, with at least two carbon double bonds. The terminology used (n/ω-) depends on the position of the first double bond from the methyl end of the molecule [5].

As PUFAs are essential fatty acids, they need to be ingested, and the most common dietary ω3-PUFAs include eicosapentaenoic acid (EPA) and docosahexaenoic acid (DHA), which originate primarily in fish oils, and alpha-linolenic acid (ALA) found in vegetable foods [4,5,6].

The first evidence of the beneficial effect of ω3-PUFAs came from pioneer studies on Eskimos in the 1970s, wherein a diet rich in ω3-PUFAs was associated with decreased coronary mortality [7]. Subsequently, substantial evidence from experimental and epidemiological studies has suggested that the intake of ω3-PUFAs could be beneficial for patients with heart failure (HF) [8].

Therefore, considering that the above mechanisms are modulators of the cardiac remodeling process, our review aims to evaluate the role of ω3-PUFAs in attenuating cardiac remodeling, with an emphasis on their antioxidant and anti-inflammatory actions.

## 2. Mechanisms through Which ω3-PUFAs Exert Their Biological Actions

**Figure 1:**
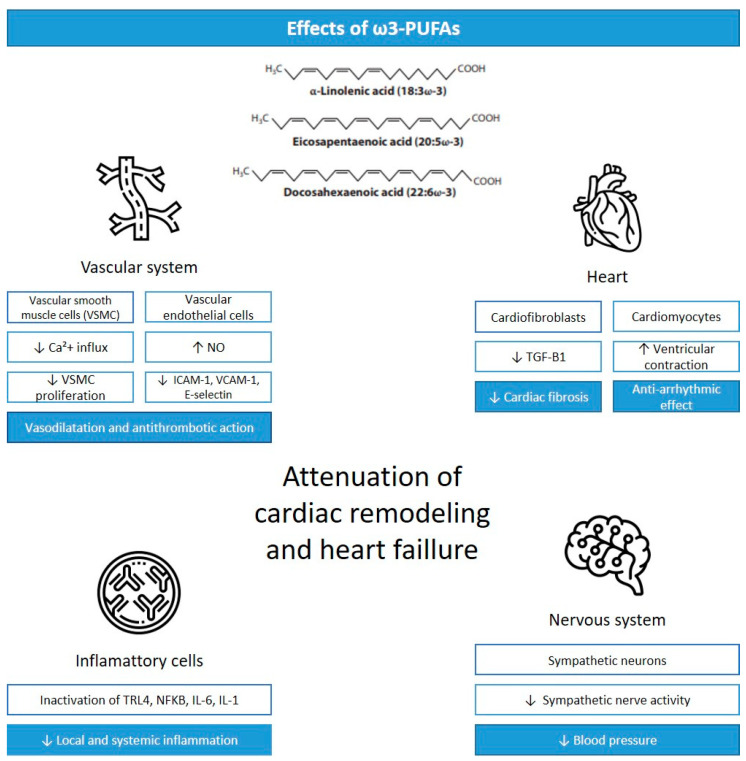
Cardiac effects of ω3-PUFAs. Legend: VSCM, vascular smooth muscle cells; Ca^+2^, calcium; NO, nitric oxide; ICAM-1, intercellular adhesion molecule-1; VCAM-1, vascular cell adhesion molecule-1; TGF-B1, transforming growth factor-β 1; TLR4, Toll-like receptor 4; NFKB, nuclear factor kappa beta; IL-6, interleukin-6; IL-1, interleukin-1; ↓ = reduction; ↑ = increase. Adapted from [14].

The health cardiac effects associated with ω3-PUFAs are mainly thought to be mediated by anti-inflammatory and antioxidant effects, alteration in cardiomyocyte mitochondrial function, the modification of cardiomyocyte ion channels, alterations in vascular endothelial response, and the modulation of autonomic nervous system activity (Figure 1) [4].

### 2.1. Anti-Inflammatory and Antioxidant Effects

The ω3-PUFAs’ anti-inflammatory and antioxidant properties can be explained by the production and antagonistic action of the arachidonic acid-derived metabolite eicosanoid and the reduced serum concentrations of pro-inflammatory cytokines, like TNF-α, IL-1, and IL-6.

In addition, ω3-PUFAs downregulate pro-inflammatory pathways, such as those of nuclear factor kappa beta (NF-kB) and NLRP3 inflammasome, and upregulate anti-inflammatory signaling pathways, including that of peroxisome proliferator-activated receptor (PPARα/γ), a transcriptional activator [4]. Eicosapentaenoic acid; DHA; and some specialized pro-resolving lipid mediators, including resolvins, protectins, and maresins, can activate nuclear factor erythropoietin 2 related factor 2 (NRF2), which has antioxidant activity, controls oxidative stress, and protects the heart from fibrosis.

The interaction between EPA and other receptors such as free fatty acid receptor 4 (Ffar4) and fibroblast GPR120 receptors appears to inhibit fibrosis [3].

Moreover, ω3-PUFAs act by reducing the sensitivity of cardiomyocytes to reactive oxygen species (ROS)-induced ischemia–reperfusion injury, increasing the levels of antioxidant enzymes superoxide dismutase (SOD) and glutathione peroxidase (GSH-Px) and acting on sirtuin1 and forkhead box protein [9].

### 2.2. Myocardial Metabolism Effects

Regarding cardiomyocyte metabolism, their effects include the ability to modify myocardial energy metabolism by changing the mitochondrial membrane phospholipid composition in cardiomyocytes, leading to decreased oxygen consumption [4].

The suppression of mitochondrial permeability transition pore (mPTP) opening, especially via DHA supplementation, prevents mitochondrial cell apoptosis [10].

### 2.3. The Effects of Cardiomyocyte Ion Channels 

Some hypotheses are used to explain the main mechanisms through which ω3-PUFAs alter the ion channels of cardiomyocytes and thus the electrophysiology of the heart. One of these hypotheses is that ion channels may contain a specific binding site for ω3-PUFAs, and when they are occupied, they can alter their function. Other hypotheses include alterations in membrane fluidity and, subsequently, ion channel function and changes in the membrane immediately surrounding the channels, which might be responsible for the ion channel effects, resulting in the slight hyperpolarization of the cell membrane, increasing the depolarizing stimuli necessary to induce an action potential [5]. Furthermore, ω3-PUFAs can affect calcium regulation, inhibiting the voltage-dependent inward calcium current during phase 2 of an action potential. Specifically, the frequency of calcium sparks, spontaneous calcium waves, and delayed after-depolarizations are reduced by ω3-PUFAs [4,11].

### 2.4. Vascular Endothelial Effects

The effects of ω3-PUFAs on the vascular endothelial response are associated with improvement in the vascular endothelial function via the activation of NO synthase, as well as the decreased expression of E-selectin, intercellular adhesion molecule-1 (ICAM-1), and vascular cell adhesion molecule-1 (VCAM-1) on endothelial cells, which are molecules related to the attachment of leucocytes to the endothelium and decreased homocysteine levels [5]. These effects are also related to the modulation of autonomic nervous system activity, increasing the vagal tone [4,5].

The antithrombotic effects of ω3-PUFAs include their ability to suppress the synthesis of platelet-derived thromboxane A2 (TXA2), which causes platelet aggregation and vasoconstriction, and to increase the plasminogen activator inhibitor-1 with a reduction in fibrinogen [4].

### 2.5. Autonomic Nervous System Effects

In the central nervous system, DHA is reported to activate NO synthase and the concentration of tetrahydrobiopterin, which may increase local NO availability and exert the tonic inhibition of the central sympathetic outflow [4]. Although the detailed mechanism remains unknown, some studies show that ω3-PUFA supplementation partially restored autonomic modulation in patients with chronic HF, associated with an improvement in arterial baroreflex function and heart rate variability, which may be related to a reduced agonist affinity of beta-receptors [5,12,13].

## 3. Experimental Evidence of the Influence of Omega-3 on Cardiac Remodeling

Numerous studies have assessed the influence of ω3-PUFA supplements on cardiac remodeling to gain insights into the mechanism of this effect. In this section, we provide an overview of the primary evidence obtained from experimental studies on the effects of ω3-PUFAs in various models of cardiac remodeling. Table 1 summarizes the significant findings of these studies.

### 3.1. Preventive Effects of ω3-PUFAs in the Development of HF

Long-term supplementation with ω3-PUFAs has been suggested to prevent cardiac remodeling in different models. A recent study compared the effects of an ALA-enriched diet to those of a non-ω3-PUFA-supplemented diet in 6-month-old rats [15]. After 12 months, ALA supplementation prevented the development of age-related diastolic dysfunction, as indicated by echocardiographic parameters such as a reduction in the E/A ratio and an increase in the isovolumetric relaxation time in the ventricular diastolic phase. Rats receiving ALA supplementation exhibited a reduction in various markers of oxidative stress (such as increased levels of SOD1, GPX1, and isocitrate dehydrogenase; a decrease in malondialdehyde (MDA) levels; and reduced levels of inflammatory markers such as NFKB1, TNF-α, and COX-2).

Another experimental study assessed the effects of ω3-PUFA supplementation over a 10-week period in rats with a specific Mn-SOD deficiency, an animal model that exhibits the typical pathophysiology of dilated cardiomyopathy [16]. The group receiving ω3-PUFA supplementation showed a significant reduction in cardiac fibrosis in comparison to rats in the control group. Additionally, the treated group exhibited a decrease in ROS production in isolated cardiomyocytes, suggesting an effect via the reduction in oxidative stress in cardiac remodeling in these rats.

Eicosapentaenoic acid supplementation was tested in a spontaneously hypertensive rat model to assess its effects on the cardiac remodeling process secondary to systemic arterial hypertension [17]. This study compared the effects of a diet containing 1.9 g/kg EPA for 20 weeks with those of a standard diet without EPA supplementation. The results showed that EPA supplementation significantly reduced the left ventricular (LV) diastolic filling pressure and interstitial collagen deposition in the LV, although it had no impact on the development of cardiac hypertrophy. This study did not assess the effects of ω3-PUFAs on oxidative stress. However, there was a substantial increase in interleukin-10 (IL-10), an anti-inflammatory cytokine, in the LV tissues of rats supplemented with EPA, suggesting the anti-inflammatory effects of this treatment.

### 3.2. Effects of ω3-PUFAs on Cardiac Remodeling Secondary to Acute MI

Acute MI is a leading cause of HF worldwide. Its complex pathophysiology involves mechanisms ranging from the release of pro-inflammatory mediators to oxidative stress in cardiomyocytes. Considering these pathophysiological processes, numerous studies have assessed the effects of ω3-PUFAs on cardiac remodeling after acute coronary ischemia [18,19,20,21,22,23,24].

Some studies suggest that prior ω3-PUFA consumption before an acute ischemic event in rat models of left anterior descending coronary artery ligation is associated with improved outcomes related to the development of HF, such as a reduction in infarct size, [20,22], a decrease in heart weight, [18,21] and a reduction in post-infarct fibrotic area [21,22]. These studies also showed reductions in inflammatory markers such as IL-1, IL-6, TGF-β, and TNF-α, [18,21,22] and a decrease in oxidative stress [20]. Rats fed with fish oil, a known source of ω3-PUFAs, for 6 weeks before infarction, showed high levels of SOD activity, although there was no increase in copper–zinc SOD and GPX activity, suggesting an antioxidative protective mechanism [20].

Supplementation with ω3-PUFAs after acute ischemic events is also associated with improved cardiovascular outcomes [18,19,21,22,23,24]. Both the continuation of ω3-PUFA supplementation in rats that were already using the supplement and the initiation of supplementation after acute infarction mitigated some manifestations of cardiac remodeling, including a reduction in fibrotic area, decreased cardiac weight, reduced arrhythmias, and reduced infarct area. An experimental study evaluating the effects of ω3-PUFA supplementation before and after infarction in rats demonstrated a reduction in mortality among rats supplemented with EPA when compared to that in rats in the control group [21]. Among the variables measured, some stood out for their relationship with oxidative stress. Fang et al. [19] demonstrated that ω3-PUFA supplementation for 1 and 12 weeks after infarction induction in rats increased the reduced levels of GSH and decreased levels of oxidized glutathione (GSSG) compared with these levels in rats fed a control diet. In addition, the study showed an increase in gamma-glutamylcysteine synthetase, an enzyme whose function is associated with GSH production, indicating a potential antioxidant effect. Another antioxidant mechanism highlighted by Wang et al. [24] is the upregulation of NRF2 and HO-1, proteins that prevent oxidative stress-related injuries. As evidenced in the study, rats that received Maresin1, a bioactive compound derived from ω3-PUFAs, after left anterior descending coronary artery ligation showed an increased expression of the NRF2/HO-1 pathway, an increase in serum levels of SOD, and a reduction in serum levels of MDA.

### 3.3. Effects of ω3-PUFAs on Pressure-Overload-Induced Cardiac Remodeling

Another experimental model of cardiac remodeling used to assess ω3-PUFA supplementation is pressure-overload-induced cardiac remodeling. In both rats subjected to transverse aortic constriction and rats subjected to abdominal aortic binding, ω3-PUFA supplementation was associated with a reduction in LV mass and cardiac fibrosis following the induction of hypertrophic cardiomyopathy [25,26,27,28,29].

Nagai et al. [25] demonstrated that supplementation with EPA for 2 weeks before and 4 weeks after transverse aortic constriction surgery reduced serum levels of both IL-6 and markers of oxidative stress, such as GPX3 and nicotinamide adenine dinucleotide phosphate oxidase (p47phox). In a similar experimental model, Dabkowski et al. [26] showed that DHA supplementation for 14 weeks was associated with alterations in mitochondrial activity, such as a reduction in the viscosity of the interfibrillar mitochondrial membranes. These changes slightly attenuated the mitochondrial permeability transition induced by ROS, an event that triggers a sequence of pathophysiological processes leading to cell death.

### 3.4. Effects of ω3-PUFAs on Cardiac Remodeling in Other HF Models

Other models of cardiac remodeling have also been employed to study ω3-PUFA supplementation. Bacova et al. and Abdellatif et al. supplemented ω3-PUFAs in rats exposed to isoproterenol, a synthetic catecholamine capable of inducing structural cardiomyopathy. The results demonstrated that the intervention was associated with a reduction in cardiac fibrosis and the remodeling of the extracellular matrix of the myocardium compared to that in the control group. Although the interventions used differed, both were associated with improved outcomes related to oxidative stress, such as increased SOD levels [30,31].

An in vitro study demonstrated positive results following DHA supplementation in a diabetic cardiomyopathy model [32]. This intervention reduced the decline in Tom20 expression, an indirect marker of mitochondrial function and quantity, in rat cardiomyocytes. Additionally, a histological evaluation of cardiac tissues showed significantly reduced ROS levels in cells treated with palmitate, an agent capable of inducing mitochondrial oxidative stress.

Olivares-Silva et al. [33] used small implanted pumps to assess the effects of resolvin-D1 infusion, a synthetic compound derived from DHA, in rats with angiotensin II-induced hypertensive cardiomyopathy. Although the oxidative stress markers were not studied in this investigation, the intervention was associated with a reduction in pro-inflammatory cytokines such as TNF-α, IL-6, IL-1β, and IL-10, and a reduction in the area of myocardial fibrosis.

### 3.5. Absence of Significant Effects of ω3-PUFAs on Cardiac Remodeling

Despite the positive results mentioned earlier, not all studies involving ω3-PUFAs have shown significant improvement in outcomes related to cardiac remodeling. In a protocol for the prevention and treatment of HF using ω3-PUFA supplementation, Galvão et al. [10] demonstrated that despite alterations in mitochondrial function and the phospholipid composition of the mitochondrial membrane, there was no increase in the survival of the studied rats. After testing ω3-PUFA supplementation in infarcted rat models, O’Shea et al. [34] also did not observe significant changes in LV function and mitochondrial respiration.

One possible explanation for the differences in results is the significant variability in methodology among studies. The interventions in these studies varied according to the type of supplement, quantity, duration, and timing of ω3-PUFA administration, making comparisons between studies challenging. Moreover, the experimental models studied and the assessed outcomes varied considerably. Therefore, further studies are necessary to better evaluate the effects of ω3-PUFAs on cardiac remodeling.

## 4. Clinical Aspects of ω3-PUFAs on HF

Interest in the cardiovascular benefits of ω3-PUFAs was initially instigated in the 1970s by Danish scientists. They concluded that Inuits (Eskimos) from northern Greenland had a remarkably low incidence of cardiovascular disease, which they attributed to a diet rich in ω3-PUFAs, consisting mainly of fish, seal meat, and whale blubber [35,36]. Subsequently, a broad scenario of ω3-PUFA benefits has been explored, such as its role in the lipid profile, endothelial integrity, arrhythmia, and thrombosis [4]. In this section, we present an overview of the results of clinical trials on the dietary ingestion and therapeutic intervention of ω3-PUFAs in cardiac remodeling and HF.

Studies on animal models have indicated that PUFA intake inhibits interstitial fibrosis and cardiac dysfunction. Food frequency studies have shown that fish consumption is inversely associated with the risk of HF [37,38,39] because fish are an important source of ω3-PUFAs. To strengthen this hypothesis, a study was conducted using a large cohort of more than 6000 participants to define the role of EPA in the primary prevention of HF. African Americans, Hispanics, Asians, and Whites from the United States, aged 45–84 years, were followed up for approximately 13 years, and their plasma EPA percentages were measured. The authors concluded that a higher plasma %EPA was associated with a lower risk of HF, including HF with reduced ejection fraction and HF with preserved ejection fraction [40].

Next, we highlight the body of clinical evidence generated by intervention studies that have been successful in demonstrating the clinical effect of ω3-PUFA supplementation on parameters compatible with cardiac remodeling. The inclusion of studies evaluating cardiovascular outcomes, in general, is beyond the scope of our review as such benefits can be achieved via the multifaceted action of ω3-PUFAs on the lipid profile, endothelium, arrhythmia, and thrombosis. The trials reviewed here are listed in Table 2 [13,41,42,43,44,45,46,47,48,49,50,51,52,53,54].

The GISSI-HF study was the first randomized clinical trial to evaluate the effect of ω3-PUFAs in patients with HF. In this study, the daily supplementation of 1 g of ω3-PUFAs for 3.9 years reduced the risk of mortality and hospitalization. In absolute terms, the reduction in the risk of mortality from all causes was 1.8% (95% CI 0.3–3.9), and mortality or hospitalization for cardiovascular reasons was 2.3% (0.0–4.6). The number needed to treat was 56 patients to prevent one death or 44 to prevent an event such as death or hospitalization for cardiovascular reasons [41]. Despite the small advantage, it is important as a simple and safe therapy. In an echocardiographic substudy of GISSHI-HF, Ghio et al. [44] demonstrated a small but statistically significant improvement in the ejection fraction.

Nodari et al. conducted two trials on ω3-PUFA supplementation. First, on a small scale, ω3-PUFA supplementation was evaluated in 44 patients with dilated cardiomyopathy. The administration of ω3-PUFAs was associated with a slight but significant improvement in parameters related to LV function and exercise capacity [42]. Subsequently, they conducted a trial with a higher dose and larger sample size. In this study, ω3-PUFA intake (2 g/day) improved LVEF by 10.4% versus a reduction of 5% with the placebo (*p* < 0.001), increased VO_2_, exercise duration, and NYHA functional class of HF with a reduction in NYHA. Additionally, hospitalization for HF was significantly suppressed in the ω3-PUFA group (6% vs. 30% in the placebo group, *p* = 0.0002).

Exploring the mechanistic pathway, a small trial in 2006 demonstrated that fish oil supplementation reduced TNF-α production in HF and improved the body weight of patients in advanced classes (NYHA III and IV) [55]. Consistent with this, subsequent studies showed that reduced inflammation and improved endothelial function [42,43,45,46,48,51] may be involved in the beneficial role of ω3-PUFAs. However, regarding laboratory markers, some studies have shown that ω3-PUFAs can decrease BNP and NT-proBNP levels [43,48,49]. The plasma concentration of BNP or NT-proBNP is an indicator of HF severity and increases exponentially as the cardiac condition worsens, with robust prognostic implications [56].

Moertl et al. [45] discussed a determining aspect of the role of ω3-PUFAs, the dose. In this randomized clinical trial, treatment with 1 to 4 g/day of ω3-PUFAs (EPA and DHA) significantly improved LVEF in a dose-dependent manner. Bernasconi et al. [57] performed a meta-analysis with more than 135,000 patients demonstrating that omega-3 supplementation with ω3-PUFAs for the prevention of cardiovascular events such as HF and MI has a greater protective effect in the presence of a higher dosage. Another meta-analysis demonstrated that the benefit of ω3-PUFAs was more significant in long-term treatment, defined as greater than 12 months [8].

Two randomized clinical trials were performed in the MI scenario and provided information on cardiac remodeling. Kalstad et al. [53] failed to detect a reduction in clinical events in elderly patients with recent MI treated with 1.8 g of ω3-PUFAs daily for 2 years, except for a reduction in combined outcomes. However, an extremely important study that consolidates evidence of the role of ω3-PUFAs in cardiac remodeling is the OMEGA-REMODEL trial [50]. In this study, a higher dose of omega-3 (4 g) was associated with a significant improvement in the remodeling parameters evaluated using magnetic resonance imaging. The LV end-systolic volume index (−5.8%, *p* = 0.017) and myocardial fibrosis without infarction (−5.6%, *p* = 0.026) were significantly suppressed in the ω3-PUFA group compared to those in the placebo group. In addition, serum biomarkers related to systemic and vascular inflammation were significantly suppressed, suggesting that the suppression of myocardial fibrosis could be one of the mechanisms of ω3-PUFA-mediated protection in the damaged myocardium.

In summary, the body of positive findings provides evidence of the role of ω3 PUFAs in critical outcomes such as mortality, a reduction in events in a combined manner, improvement in NYHA functional class and functional capacity, and systolic and diastolic function parameters compatible with the attenuation of remodeling. In addition, differences in prognostic laboratory markers such as BNP and mechanistic markers such as inflammatory markers were observed.

However, we would also like to highlight some studies that did not demonstrate significant beneficial effects of ω3-PUFA supplementation. A small trial conducted in 2001 evaluated the effects of omega-3 supplementation for 12 weeks post-MI. Left ventricular function and atrial natriuretic peptide concentrations did not differ, but the number of patients in this evaluation was small (N = 55) [58]. Another study conducted in 2006 on patients post-infarction with an even smaller N (N = 25) did not show improvement in the ejection fraction or the functional class of patients with HF; only an improvement in baroreflex function was observed [13]. Subsequently, a large study, the Alpha Omega Trial, which included 4837 patients, showed that ω3-PUFAs at low dosage did not significantly reduce cardiovascular complications in patients post-MI; however, cardiac function was not evaluated in this study [59].

An important debate that can help to better understand and define the role of omega-3 is the offered omega-3 formulation. As observed above, isolated EPA and DHA or their combination constituted the main formulation. However, different results were obtained using the proposed formulations. Eicosapentaenoic acid and DHA differ in their molecular structures and exert distinct biological effects. For example, EPA results in E-resolvins, whereas DHA results in D-resolvins, with differences in oxidation. Eicosapentaenoic acid may stabilize cholesterol membrane rafts, whereas DHA may not [60,61]. Therefore, a mixture of EPA and DHA or a comparison of the use of different compounds may not provide complete information. In a recent study among patients with high triglyceride levels despite statin use (REDUCE-IT), the risk of ischemic events, including cardiovascular death, was significantly lower among those who received 2 g of icosapent ethyl (an ultrapure product of EPA) twice daily than among those who received the placebo [62].

In short, clinical and experimental studies indicate that the attenuation of cardiac remodeling and myocardial fibrosis may be one of the protective pathways promoted by ω3-PUFAs for improvement in systolic and diastolic function as well as relevant clinical outcomes. Given the importance of HF as a pathological entity with high morbidity and mortality rates despite current therapies, measures to slow or reverse remodeling are always highly desired. Although the ideal time of use, whether in preventive or curative cardiology, as well as the formulation, dose, and duration of use, remain uncertain in HF, the available data suggest that the highly positive results of dietary supplementation are mainly related to the duration and dosage of the treatment. The key to the future of ω3-PUFAs is perhaps no longer the evaluation of EPA and DHA as equivalents or a combined supplement but a better understanding of their individual roles as distinct molecules and perhaps of their use as ultra-pure compounds.

There is a discrepancy between the remarkable results obtained using ω3-PUFAs in experimental and clinical settings. A recent article that specifically addresses the justifications for this discrepancy highlights interesting aspects to consider [63]. First, a critical point of translational medicine is that the experimental conditions used in animal studies are more homogeneous and rigorously controlled than in human studies. The bioavailability of omega 3 varies according to the different forms of these fatty acids ingested. Defining the appropriate dose for use in humans based on animal studies is a difficult task. The effectiveness of omega 3 is influenced by the intestinal microbiota and also depends on the non-lipid components of the diet. Furthermore, different individuals may present a diverse degree of response to LC-omega-3 PUFA dietary supplements, suggesting that such heterogeneity may be justified by genetic or epigenetic variability. Some human studies have begun to use the strategy of subdividing participants into omega-3 PUFA responders and omega-3 PUFA non-responders to understand the benefit of this supplement better. Finally, we highlight that experimental benefits generally comprise molecular assessment models to support a pathophysiological explanation, while clinical studies have been disappointing when strict parameters (such as mortality) have been used to evaluate the efficacy of ω3-PUFAs in clinical scenarios.

In conclusion, despite the actual evidence of the beneficial effects of ω3-PUFAs in different scenarios, large randomized studies specifically addressing ω3-PUFAs and cardiac remodeling remain lacking. Therefore, we cannot recommend supplementation to prevent or mitigate cardiac remodeling. However, while awaiting further studies, we believe that encouraging fish intake could be an attractive strategy.

## Figures and Tables

**Table 1:** Experimental evidence of inflammation and oxidative stress in cardiac remodeling.

Author, Year	Model	ω3-PUFA	Cardiac Remodeling Effect	Mechanisms
Saeedi, 2023 [15]	Mice (aged)	diet containing 7.3% ALA for 12 months	prevents diastolic dysfunction (↓ E/A, ↑ IVRT) ↓ ECM remodeling	↓ inflammation (↓ NFKB1, ↓ TNF-α, ↓ COX2), ↓ OS (↓ MDA, ↑ SOD, ↑ GPX), ↓ apoptosis
Li, 2017 [16]	Mice (heart/muscle-specific Mn-SOD-deficient)	3 mg/(kg·day) n-3 PUFA for 10 weeks	↓ Fibrosis	↓ ROS, ↓ protein carbonylation, ↓ apoptosis
Gharrae, 2022 [17]	Rats (hypertension)	EPA 1.9 g/kg for 20 weeks	↓ E/e′, ↓ fibrosis	↓ inflammation (↑ IL-10)
Fosshaug, 2011 [18]	Rats (MI)	krill oil (EPA + DHA 0.75% of energy intake) 14 days before MI until 7 weeks after	↓ HW, ↓ LVDD, ↓ RWT	↓ ANP, TGF-β, TNF-α, IL-1, IL-6, MCP-1
Fang, 2011 [19]	Rats (MI)	PUFA (450 mg/kg (30% ALA) 12 weeks	↓ LVDD, ↓ LVSD, ↑ FS, ↓ LVWt, ↑ LV dP/dt, ↑ LVESPVR ↓ LVEDPVR ↓ LVEDP ↓ Tau, ↓ fibrosis (collagen)	↓ OS (↑ GSH total and reduced, ↓ GSSG)
Abdukevum, 2016 [20]	Rats (MI)	fish oil (n-3 PUFA); sunflower seed oil (n-6 PUFA); or beef tallow (saturated fat, SF) for 6 weeks before MI	n-3 PUFA ↓ Infarct size	n-3 PUFA ↓ LPO, MDA, ↑ SOD
Takamura, 2017 [21]	Rats (MI)	EPA 28 days pre and 28 post-MI, 1 g/(kg·day)	↓ mortality, ↓ heart weight, ↓ LVEDD, ↓ LVESD, ↑ LVEF, ↑ %FS, ↓ CSA, ↓ fibrosis, ↓ ANP and BNP mRNA expression	↓ macrophage polarization ↓ inflammation (↓ TGFB, CCL2, EMR1, IL-6, IRF5 ↑ MRC1, VEGF),
Parikh, 2019 [22]	Rats (MI)	Flaxseed (milled and oil) 2 weeks pre and 8 weeks post-MI.	↓ MI size, ↓ arrhythmias, ↓ LVID, ↓ fibrosis	↓ inflammation (TNF-α)
Habicht, 2020 [23]	Rats (MI)	DHA 0.26 g/kg for 7 days after MI	↓ LVEDP, ↑ EF, ↑ dP/dt, ↓ Tau	↓ inflammation (↓ TNF-α, IL-1, IL-10, ↓ chemokine mRNA), ↓ OS (↓ GPX, ↓ HO-1, variation in UCP3)
Wang, 2022 [24]	Rats (MI)	Maresin1 (intraperitoneal injection 10 ng/g once every 2 days for 28 days)	↑ EF, ↑ FS, ↓ LVEDV, ↓ LVESV, ↓ LVIDD, ↓ LVIDS, ↓ fibrosis (↓ collagen, ↓ α-SMA),	↓ OS (↑NRF2/HO-1, ↑ SOD, ↓ MDA), ↓ inflammation (↓ TLR4, TNFa, IL-6), ↓ apoptosis (↓ Bax, caspase-3, ↑ Bcl2)
Nagai, 2013 [25]	Rats (transverse aortic constriction)	EPA (7% of the total energy) 2 weeks pre and 4 weeks post TAC	↓ HW, ↓ LVEDD,↓ LVESD, ↓ LVEDP, ↑ FS, ↓ AWT, ↓ PWT, ↓ CSA, ↓ fibrosis (↓ TGF-β)	↓ inflammation (IL6), ↓ OS (GPX, p47 phox)
Dabkowski, 2013 [26]	Rats (transverse aortic constriction)	DHA 2.3% of energy intake, 3 days after surgery for 14 weeks	↑ FS, ↑ EF	↓ ROS-induced mitochondrial permeability transition
Toko, 2020 [27]	Rats (transverse aortic constriction)	EPA + DHA, 1.5 mg/g for 4 weeks	↓ LVESD, ↑ FS, ↓ HW, ↓ CSA, ↓ Fibrosis	↓ inflammation (↓ leukocytes and macrophages, ↓ TNFα and MIP-1α), no difference in MDA and iso-PGF2α
Shah, 2009 [28]	Rats (abdominal aortic banding)	EPA + DHA 2.3% of energy intake,	↓ LVM	↓ inflammation (↓ AA in cardiac phospholipids, ↓ TA2 excretion)
Duda, 2009 [29]	Rats (abdominal aortic banding)	EPA + DHA or ALA 0.7–7% of energy intake, 1 week before and 12 weeks after surgery	↑ FS, ↑ EF, ↓ PWT, ↓ LVEDV, ↓ LVESV	↓ inflammation (↓ AA in cardiac phospholipids, ↓ TNF-α, ↓ TA2, ↓ 6-KPGF1α)
Szeiffova, 2020 [30]	Rats (isoproterenol)	Omega-3 1.68 g/(kg·day) until 60 days	↓ fibrosis and ECM remodeling, ↓ myocardial injury ↓ LVW	↓ OS (↑ SOD)
Abdellatif, 2023 [31]	Rats (isoproterenol)	Calanus oil 400 mg/kg b.wt for 4 weeks	↓ LVPWd, ↓ IVd, ↑ LVIDD, ↓ IVs, ↑ LVIDS, ↓ LVM, ↑FS ↑ EF, ↓ HW, ↓ hypertrophy	↓ MDA, ↑TAC
Gui, 2020 [32]	Cardiomyocytes	DHA (20 μM) for 16 weeks	↓ HW, ↓ hypertrophy, ↓ fibrosis (↓ α-SMA)	↓ OS (↓ Tom20, ↓ R OS, ↓ roGFP, ↓ 4HNE), ↓ apoptosis
Olivares-Silva, 2021 [33]	Rats (ang II hypertension)	RvD1 (3 μg/(kg·day) i.p.) after surgery until euthanized	↓ CSA, ↓ IVSWT, ↓ PWT, ↓ LVEDD, ↓ fibrosis	↓ inflammation (↓ granulation, neutrophil and macrophage; ↓ ICAM-1, VCAM-1, IL-1β, TNF-α, IL-6, IL-10, KC e MCP-1)

Legend: MI, myocardial infarction; EPA, eicosapentaenoic acid; LV, left ventricle; LVEDD, LV end-diastolic diameter; LVESD, LV end-systolic diameter; EF, ejection fraction; FS, fractional shortening; CSA, cross-sectional area; TGF-β, transforming growth factor-β; CCL2, chemokine C-C motif ligand 2; EMR1, EGF-like module-containing mucin-like hormone receptor-like 1; IL, interleukin; IRF5, interferon regulatory factor 5; MRC1, mannose receptor C type 1; VEGF, vascular endothelial growth factor; ANP, atrial natriuretic peptide; BNP, B-type natriuretic peptide; LVID, LV internal diameter; TNF-α, tumor necrosis factor alpha; ALA, alpha-lipoic acid; IVRT, isovolumic relaxation time; NF-κB, nuclear factor kappa beta; COX2, cyclooxygenase2; OS, oxidative stress; MDA, malondialdehyde; SOD, superoxide dismutase; GPX, glutathione peroxidase; ECM, extracellular matrix; LVW, LV weight; LVIDD, LV internal diastolic diameter; LVISD, LV internal systolic diameter; αSMA, smooth muscle alpha-actin; LVEDV, LV end-diastolic volume; LVESV, LV end-systolic volume; NRF2, nuclear factor erythroid-derived 2-related factor 2; HO-1, heme oxygenase-1; TLR4, Toll-like receptor 4; BAX, BCL-2 associated protein X; IL-10, interleukin-10; PUFA, polyunsaturated fatty acids; ALA, alpha-linolenic acid; LVDD, LV diastolic diameter; LVSD, LV systolic diameter; dP/dt, ratio of pressure change in the ventricular cavity during the isovolemic contraction period; LVESPDR, LV end-systolic pressure volume relation; LVEDPVR, LV end-diastolic pressure volume relation; LVEDP, LV end-diastolic pressure; GSH, reduced glutathione; GSSG, oxidized glutathione; HW, heart weight; AWT, anterior wall thickness; PWT, posterior wall thickness; p47phox, 47-kDa α-subunit of nicotinamide adenine dinucleotide phosphate oxidase; Tom20, anti-translocase of outer membrane 20; ROS, reactive oxygen species; roGFP, reduction–oxidation sensitive green fluorescent protein; 4-HNE, anti-4-hydroxynonenal; UCP3, uncoupling protein 3; LVPWd, end-diastolic left ventricular posterior wall thickness; IVd, interventricular septum in end diastole; IVs, interventricular septum in end systole; LVM, left ventricular mass; TAC, total antioxidant capacity; RvD1, resolvin-D1; IVWST, interventricular septal wall thickness; ICAM1, intercellular adhesion molecule-1; VCAM-1, vascular cell adhesion molecule-1; KC, keratinocyte-derived chemokine; MCP1, monocyte chemoattractant protein-1; MIP-1α, macrophage inflammatory protein-1 alpha; PGF2α, prostaglandin F2α; RWT, relative wall thickness; AA, arachidonic acid, TA2, thromboxane A2; 6-KPGF1α, 6-keto prostaglandin F1α; LPO, lipid hydroperoxides; ↓ = reduction, ↑ = increase.

**Table 2:** Clinical trials with positive findings of omega-3 on cardiac remodeling.

Author, Year	Country	N	Patients	Omega-3 Dose/Day	Follow Up	Main Indexes
Tavazzi, 2008 [41]	Italy	7046	CHF NYHA II–IV	1 g (EPA/DHA)	3.9 years	↓ Mortality (all causes and owing to CV), ↓ hospitalization
Nodari, 2009 [42]	Italy	44	CHF by DCM EF ≤ 45%	1 g (EPA/DHA)	6 months	↓ LVESV, ↑ EF, ↓ arrhythmia risk, ↑ VO_2_, ↓ serum norepinephrine TNFα, IL-1, IL-6
Zhao, 2009 [43]	China	76	CHF NYHA II–III DCM, EF < 40%	2 g (EPA/DHA)	3 months	↓ CRP, ↓ TNFα, ↓ IL6, ↓ ICAM-1, ↓ NT-proBNP
Ghio, 2010 [44]	Italy	608	Patients with CHF NYHA II–IV ^1^	1 g (EPA/DHA)	3 years	↑ EF
Moertl, 2011 [45]	Austria	43	CHF NYHA III–IV DCM EF < 35%	1–4 g (EPA/DHA)	3 months	↑ EF, ↓ IL-6
Nodari, 2011 [46]	Italy, USA	133	CHF NYHA II–III DCM EF ≤ 45%	2 g (EPA/DHA)	12 months	↑ EF, ↑ VO_2_, ↑ exercise duration, ↓ mean NYHA class, ↓ hospitalization, improved systolic and diastolic function, ↓ IL-6, IL-1, TNFα
Kojuri, 2013 [47]	Iran	70	CHF NYHA II–III ICM EF < 40%	2 g (EPA/DHA)	6 months	↓ Tei index, ↑ AM index
Kohashi, 2014 [48]	Japan	139	CHF mean EF 37.6% ± 8%	1.8 g (EPA/DHA)	12 months	↓ BNP, ↓ BP, ↓ cholesterol, ↑ EF, ↓ CRP, ↓ mean NYHA class, ↓ MCP-1, ↓ TNFα, ↓ AA
Chrysohoou, 2016 [49]	Greece	205	CHF NYHA I–III ICM and DCM	1 g (EPA/DHA)	6 months	↓ BNP, ↓ LVESD, ↓ LVEDD, ↓ LA, ↓ Etv/Atv
Heydari et al., 2016 [50]	EUA	358	MI	4 g (EPA/DHA)	6 months	↓ LVESVI, ↓ Fibrosis
Oikonomou et al., 2019 [51]	Greece	31	CHF–ICM with EF < 40%	2 g (EPA/DHA)	6 weeks	↑ EF, ↓ global longitudinal strain, ↓ E/e’ ratio, ↓ ST2 levels, ↑ flow-mediated dilation, ↓ CRP levels
Djoussé et al., 2020 [52]	EUA	499	patients with CHF ^2^	1 g (EPA/DHA)	5.3 years	↓ recurrent CHF hospitalization
Kalstad et al., 2021 [53]	Norway	1027	MI	1.8 g (EPA/DHA)	2 years	↓ composite nonfatal MI, CRev, stroke, all-cause death, CHF hospitalization.

Legends: ↓ = reduction; ↑ = increase; DCM, dilated cardiomyopathy; EF, ejection fraction; EPA, eicosapentaenoic acid; DHA, docosahexaenoic acid; LV, left ventricle; LVESV, LV end-systolic volume; TNF-α, tumor necrosis factor alpha; IL, interleukin; CHF, congestive heart failure NYHA, New York Heart Association; MI, myocardial infarction; CRP, C-reactive protein; ICAM1, intercellular adhesion molecule-1; NT-proBNP, aminoterminal pro-B-type natriuretic peptide; VO_2_, oxygen uptake; ICM, ischemic cardiomyopathy; AM index, late diastolic velocity index; BP, blood pressure; MCP-1, monocyte chemoattractant protein-1; AA, arachidonic acid; BNP, B-type natriuretic peptide; LVESD, LV end-systolic diameter; LVEDD, LV end-diastolic diameter; LA, left atrium; Etv/Atv, ratio of E and A waves of tricuspid annulus; CV, cardiovascular; LVESVI, left ventricular systolic volume index; TG, triglycerides; CRev, coronary revascularization; UA, unstable angina; ST2, suppression of tumorigenicity 2. ^1^ Substudy: effects of n-3 polyunsaturated fatty acids and rosuvastatin on left ventricular function in chronic heart failure: a substudy of the GISSI-HF trial; ^2^ subanalysis from VITAL: vitamin D supplements and prevention of cancer and cardiovascular disease.

## Data Availability

No new data were created or analyzed in this study. Data sharing is not applicable to this article.

## References

[1] Azevedo P.S., Polegato B.F., Minicucci M.F., Paiva S.A.R., Zornoff L.A.M. (2016). Cardiac Remodeling: Concepts, Clinical Impact, Pathophysiological Mechanisms and Pharmacologic Treatment. Arq. Bras. Cardiol..

[2] Martins D., Garcia L.R., Queiroz D.A.R., Lazzarin T., Tonon C.R., Balin P.D.S., Polegato B.F., de Paiva S.A.R., Azevedo P.S., Minicucci M.F. (2022). Oxidative Stress as a Therapeutic Target of Cardiac Remodeling. Antioxidants.

[3] Oppedisano F., Mollace R., Tavernese A., Gliozzi M., Musolino V., Macrì R., Carresi C., Maiuolo J., Serra M., Cardamone A. (2021). PUFA Supplementation and Heart Failure: Effects on Fibrosis and Cardiac Remodeling. Nutrients.

[4] Sakamoto A., Saotome M., Iguchi K., Maekawa Y. (2019). Marine-Derived Omega-3 Polyunsaturated Fatty Acids and Heart Failure: Current Understanding for Basic to Clinical Relevance. Int. J. Mol. Sci..

[5] Aarsetoey H., Grundt H., Nygaard O., Nilsen D.W.T. (2012). The Role of Long-Chained Marine N-3 Polyunsaturated Fatty Acids in Cardiovascular Disease. Cardiol. Res. Pract..

[6] O’Connell T.D., Block R.C., Huang S.P., Shearer G.C. (2017). ω3-Polyunsaturated fatty acids for heart failure: Effects of dose on efficacy and novel signaling through free fatty acid receptor 4. J. Mol. Cell Cardiol..

[7] Bang H.O., Dyerberg J. (1980). Lipid metabolism and ischemic heart disease in Greenland Eskimos. Adv. Nutri. Res..

[8] Wang C., Xiong B., Huang J. (2016). The Role of Omega-3 Polyunsaturated Fatty Acids in Heart Failure: A Meta-Analysis of Randomised Controlled Trials. Nutrients.

[9] Oppedisano F., Macrì R., Gliozzi M., Musolino V., Carresi C., Maiuolo J., Bosco F., Nucera S., Caterina Zito M., Guarnieri L. (2020). The Anti-Inflammatory and Antioxidant Properties of n-3 PUFAs: Their Role in Cardiovascular Protection. Biomedicines.

[10] Galvao T.F., Khairallah R.J., Dabkowski E.R., Brown B.H., Hecker P.A., O’Connell K.A., O’Shea K.M., Sabbah H.N., Rastogi S., Daneault C. (2013). Marine n3 polyunsaturated fatty acids enhance resistance to mitochondrial permeability transition in heart failure but do not improve survival. Am. J. Physiol. Heart Circ. Physiol..

[11] Richardson E.S., Iaizzo P.A., Xiao Y.F. (2011). Electrophysiological Mechanisms of the Anti-arrhythmic Effects of Omega-3 Fatty Acids. J. Cardiovasc. Trans. Res..

[12] La Rovere M.T., Staszewsky L., Barlera S., Maestri R., Mezzani A., Midi P., Marchioli R., Maggioni A.P., Tognoni G., Tavazzi L. (2013). n-3PUFA and Holter-derived autonomic variables in patients with heart failure: Data from the Gruppo Italiano per lo Studio della Sopravvivenza nell’Insufficienza Cardiaca (GISSI-HF) Holter substudy. Heart Rhythm..

[13] Radaelli A., Cazzaniga M., Viola A., Balestri G., Janetti M.B., Signorini M.G., Castiglioni P., Azzellino A., Mancia G., Ferrari A.U. (2006). Enhanced Baroreceptor Control of the Cardiovascular System by Polyunsaturated Fatty Acids in Heart Failure Patients. J. Am. Coll. Cardiol..

[14] Djuricic I., Calder P.C. (2023). Pros and Cons of Long-Chain Omega-3 Polyunsaturated Fatty Acids in Cardiovascular Health. Annu. Rev. Pharmacol. Toxicol..

[15] Saeedi Saravi S.S., Bonetti N.R., Vukolic A., Vdovenko D., Lee P., Liberale L., Basso C., Rizzo S., Akhmedov A., Lüscher T.F. (2023). Long-term dietary n3 fatty acid prevents aging-related cardiac diastolic and vascular dysfunction. Vascul Pharmacol..

[16] Li Q., Yu Q., Na R., Liu B. (2017). Omega-3 polyunsaturated fatty acids prevent murine dilated cardiomyopathy by reducing oxidative stress and cardiomyocyte apoptosis. Exp. Ther. Med..

[17] Gharraee N., Wang Z., Pflum A., Medina-Hernandez D., Herrington D., Zhu X., Meléndez G.C. (2022). Eicosapentaenoic Acid Ameliorates Cardiac Fibrosis and Tissue Inflammation in Spontaneously Hypertensive Rats. J. Lipid Res..

[18] Fosshaug L.E., Berge R.K., Beitnes J.O., Berge K., Vik H., Aukrust P., Gullestad L., Vinge L.E., Øie E. (2011). Krill oil attenuates left ventricular dilatation after myocardial infarction in rats. Lipids Health Dis..

[19] Fang Y., Favre J., Vercauteren M., Laillet B., Remy-Jouet I., Skiba M., Lallemand F., Dehaudt C., Monteil C., Thuillez C. (2011). Reduced cardiac remodelling and prevention of glutathione deficiency after omega-3 supplementation in chronic heart failure. Fundam. Clin. Pharmacol..

[20] Abdukeyum G.G., Owen A.J., Larkin T.A., McLennan P.L. (2016). Up-Regulation of Mitochondrial Antioxidant Superoxide Dismutase Underpins Persistent Cardiac Nutritional-Preconditioning by Long Chain n-3 Polyunsaturated Fatty Acids in the Rat. J. Clin. Med..

[21] Takamura M., Kurokawa K., Ootsuji H., Inoue O., Okada H., Nomura A., Kaneko S., Usui S. (2017). Long-Term Administration of Eicosapentaenoic Acid Improves Post-Myocardial Infarction Cardiac Remodeling in Mice by Regulating Macrophage Polarization. J. Am. Heart Assoc..

[22] Parikh M., Raj P., Austria J.A., Yu L., Garg B., Netticadan T., Pierce G.N. (2019). Dietary flaxseed protects against ventricular arrhythmias and left ventricular dilation after a myocardial infarction. J. Nutr. Biochem..

[23] Habicht I., Mohsen G., Eichhorn L., Frede S., Weisheit C., Hilbert T., Treede H., Güresir E., Dewald O., Duerr G.D. (2020). DHA Supplementation Attenuates MI-Induced LV Matrix Remodeling and Dysfunction in Mice. Oxid. Med. Cell Longev..

[24] Wang F., Gong Y., Chen T., Li B., Zhang W., Yin L., Zhao H., Tang Y., Wang X., Huang C. (2022). Maresin1 ameliorates ventricular remodelling and arrhythmia in mice models of myocardial infarction via NRF2/HO-1 and TLR4/NF-kB signalling. Int. Immunopharmacol..

[25] Nagai T., Anzai T., Mano Y., Kaneko H., Anzai A., Sugano Y., Maekawa Y., Takahashi T., Yoshikawa T., Fukuda K. (2013). Eicosapentaenoic acid suppresses adverse effects of C-reactive protein overexpression on pressure overload-induced cardiac remodeling. Heart Vessels..

[26] Dabkowski E.R., O’Connell K.A., Xu W., Ribeiro R.F., Hecker P.A., Shekar K.C., Daneault C., Des Rosiers C., Stanley W.C. (2013). Docosahexaenoic acid supplementation alters key properties of cardiac mitochondria and modestly attenuates development of left ventricular dysfunction in pressure overload-induced heart failure. Cardiovasc. Drugs Ther..

[27] Toko H., Morita H., Katakura M., Hashimoto M., Ko T., Bujo S., Adachi Y., Ueda K., Murakami H., Ishizuka M. (2020). Omega-3 fatty acid prevents the development of heart failure by changing fatty acid composition in the heart. Sci. Rep..

[28] Shah K.B., Duda M.K., O’Shea K.M., Sparagna G.C., Chess D.J., Khairallah R.J., Robillard-Frayne I., Xu W., Murphy R.C., Des Rosiers C. (2009). The cardioprotective effects of fish oil during pressure overload are blocked by high fat intake: Role of cardiac phospholipid remodeling. Hypertens.

[29] Duda M.K., O’Shea K.M., Tintinu A., Xu W., Khairallah R.J., Barrows B.R., Chess D.J., Azimzadeh A.M., Harris W.S., Sharov V.G. (2009). Fish oil, but not flaxseed oil, decreases inflammation and prevents pressure overload-induced cardiac dysfunction. Cardiovasc. Res..

[30] Szeiffova Bacova B., Viczenczova C., Andelova K., Sykora M., Chaudagar K., Barancik M., Adamcova M., Knezl V., Egan Benova T., Weismann P. (2020). Antiarrhythmic Effects of Melatonin and Omega-3 Are Linked with Protection of Myocardial Cx43 Topology and Suppression of Fibrosis in Catecholamine Stressed Normotensive and Hypertensive Rats. Antioxidants.

[31] Abdellatif S.Y., Fares N.H., Elsharkawy S.H., Mahmoud Y.I. (2023). Calanus oil attenuates isoproterenol-induced cardiac hypertrophy by regulating myocardial remodeling and oxidative stress. Ultrastruct. Pathol..

[32] Gui T., Li Y., Zhang S., Zhang N., Sun Y., Liu F., Chen Q., Gai Z. (2020). Docosahexaenoic acid protects against palmitate-induced mitochondrial dysfunction in diabetic cardiomyopathy. Biomed. Pharmacother..

[33] Olivares-Silva F., De Gregorio N., Espitia-Corredor J., Espinoza C., Vivar R., Silva D., Osorio J.M., Lavandero S., Peiró C., Sánchez-Ferrer C. (2021). Resolvin-D1 attenuation of angiotensin II-induced cardiac inflammation in mice is associated with prevention of cardiac remodeling and hypertension. Biochim. Biophys. Acta Mol. Basis Dis..

[34] O’Shea K.M., Khairallah R.J., Sparagna G.C., Xu W., Hecker P.A., Robillard-Frayne I., Des Rosiers C., Kristian T., Murphy R.C., Fiskum G. (2009). Dietary ω-3 fatty acids alter cardiac mitochondrial phospholipid composition and delay Ca2+-induced permeability transition. J. Mol. Cell Cardiol..

[35] Dyerberg J., Bang H.O., Stoffersen E., Moncada S., Vane J.R. (1978). Eicosapentaenoic acid and prevention of thrombosis and atherosclerosis?. Lancet Lond Engl..

[36] Dyerberg J., Bang H.O. (1979). Haemostatic function and platelet polyunsatured fatty acids in eskimos. Lancet.

[37] Mozaffarian D., Bryson C.L., Lemaitre R.N., Burke G.L., Siscovick D.S. (2005). Fish Intake and Risk of Incident Heart Failure. J. Am. Coll. Cardiol..

[38] Belin R.J., Greenland P., Martin L., Oberman A., Tinker L., Robinson J., Larson J., Van Horn L., Lloyd-Jones D. (2011). Fish Intake and the Risk of Incident Heart Failure: The Women’s Health Initiative. Circ. Heart Fail..

[39] Wilk J.B., Tsai M.Y., Hanson N.Q., Gaziano J.M., Djoussé L. (2012). Plasma and dietary omega-3 fatty acids, fish intake, and heart failure risk in the Physicians’ Health Study. Am. J. Clin. Nutr..

[40] Block R.C., Liu L., Herrington D.M., Huang S., Tsai M.Y., O’Connell T.D., Shearer G.C. (2019). Predicting Risk for Incident Heart Failure With Omega-3 Fatty Acids. JACC Heart Fail..

[41] Tavazzi L., Maggioni A.P., Marchioli R., Barlera S., Franzosi M.G., Latini R., Lucci D., Nicolosi G.L., Porcu M., Tognoni G. (2008). Gissi-HF Investigators. Effect of n-3 polyunsaturated fatty acids in patients with chronic heart failure (the GISSI-HF trial): A randomised, double-blind, placebo-controlled trial. Lancet.

[42] Nodari S., Metra M., Milesi G., Manerba A., Cesana B.M., Gheorghiade M., Dei Cas L. (2009). The role of n-3 PUFAs in preventing the arrhythmic risk in patients with idiopathic dilated cardiomyopathy. Cardiovasc. Drugs Ther..

[43] Zhao Y.T., Shao L., Teng L.L., Hu B., Luo Y., Yu X., Zhang D.F., Zhang H. (2009). Effects of n-3 polyunsaturated fatty acid therapy on plasma inflammatory markers and N-terminal pro-brain natriuretic peptide in elderly patients with chronic heart failure. J. Int. Med. Res..

[44] Ghio S., Scelsi L., Latini R., Masson S., Eleuteri E., Palvarini M., Vriz O., Pasotti M., Gorini M., Marchioli R. (2010). Effects of n-3 polyunsaturated fatty acids and of rosuvastatin on left ventricular function in chronic heart failure: A substudy of GISSI-HF trial. Eur. J. Heart Fail..

[45] Moertl D., Hammer A., Steiner S., Hutuleac R., Vonbank K., Berger R. (2011). Dose-dependent effects of omega-3-polyunsaturated fatty acids on systolic left ventricular function, endothelial function, and markers of inflammation in chronic heart failure of nonischemic origin: A double-blind, placebo-controlled, 3-arm study. Am. Heart J..

[46] Nodari S., Triggiani M., Campia U., Manerba A., Milesi G., Cesana B.M., Gheorghiade M., Dei Cas L. (2011). Effects of n-3 polyunsaturated fatty acids on left ventricular function and functional capacity in patients with dilated cardiomyopathy. J. Am. Coll. Cardiol..

[47] Kojuri J., Ostovan M.A., Rezaian G.R., Archin Dialameh P., Zamiri N., Sharifkazemi M.B., Jannati M. (2013). Effect of omega-3 on brain natriuretic peptide and echocardiographic findings in heart failure: Double-blind placebo-controlled randomized trial. J. Cardiovasc. Dis. Res..

[48] Kohashi K., Nakagomi A., Saiki Y., Morisawa T., Kosugi M., Kusama Y., Atarashi H., Shimizu W. (2014). Effects of Eicosapentaenoic Acid on the Levels of Inflammatory Markers, Cardiac Function and Long-Term Prognosis in Chronic Heart Failure Patients with Dyslipidemia. J. Atheroscler. Thromb..

[49] Chrysohoou C., Metallinos G., Georgiopoulos G., Mendrinos D., Papanikolaou A., Magkas N., Pitsavos C., Vyssoulis G., Stefanadis C., Tousoulis D. (2016). Short term omega-3 polyunsaturated fatty acid supplementation induces favorable changes in right ventricle function and diastolic filling pressure in patients with chronic heart failure; A randomized clinical trial. Vascul Pharmacol..

[50] Heydari B., Abdullah S., Pottala J.V., Shah R., Abbasi S., Mandry D., Francis S.A., Lumish H., Ghoshhajra B.B., Hoffmann U. (2016). Effect of Omega-3 Acid Ethyl Esters on Left Ventricular Remodeling After Acute Myocardial Infarction: The OMEGA-REMODEL Randomized Clinical Trial. Circulation.

[51] Oikonomou E., Vogiatzi G., Karlis D., Siasos G., Chrysohoou C., Zografos T., Lazaros G., Tsalamandris S., Mourouzis K., Georgiopoulos G. (2019). Effects of omega-3 polyunsaturated fatty acids on fibrosis, endothelial function and myocardial performance, in ischemic heart failure patients. Clin. Nutr. Edinb. Scotl..

[52] Djoussé L., Cook N.R., Kim E., Bodar V., Walter J., Bubes V., Luttmann-Gibson H., Mora S., Joseph J., Lee I.M. (2020). Supplementation With Vitamin D and Omega-3 Fatty Acids and Incidence of Heart Failure Hospitalization: VITAL-Heart Failure. Circulation.

[53] Kalstad A.A., Myhre P.L., Laake K., Tveit S.H., Schmidt E.B., Smith P., Nilsen D.W.T., Tveit A., Fagerland M.W., Solheim S. (2021). Effects of n-3 Fatty Acid Supplements in Elderly Patients After Myocardial Infarction: A Randomized, Controlled Trial. Circulation.

[54] Selvaraj S., Bhatt D.L., Steg P.G., Miller M., Brinton E.A., Jacobson T.A., Juliano R.A., Jiao L., Tardif J.C., Ballantyne C.M. (2022). REDUCE-IT Investigators. Impact of Icosapent Ethyl on Cardiovascular Risk Reduction in Patients With Heart Failure in REDUCE-IT. J. Am. Heart Assoc..

[55] Mehra M.R., Lavie C.J., Ventura H.O., Milani R.V. (2006). Fish oils produce anti-inflammatory effects and improve body weight in severe heart failure. J. Heart Lung Transplant. Off. Publ. Int. Soc. Heart Transplant..

[56] Buchan T.A., Ching C., Foroutan F., Malik A., Daza J.F., Hing N.N.F., Siemieniuk R., Evaniew N., Orchanian-Cheff A., Ross H.J. (2022). Prognostic value of natriuretic peptides in heart failure: Systematic review and meta-analysis. Heart Fail. Rev..

[57] Bernasconi A.A., Wiest M.M., Lavie C.J., Milani R.V., Laukkanen J.A. (2021). Effect of Omega-3 Dosage on Cardiovascular Outcomes. Mayo Clin. Proc..

[58] Skou H.A., Toft E., Christensen J.H., Hansen J.B., Dyerberg J., Schmidt E.B. (2001). N-3 Fatty Acids and Cardiac Function after Myocardial Infarction in Denmark. Int. J. Circumpolar Health.

[59] Kromhout D., Giltay E.J., Geleijnse J.M. (2010). n–3 Fatty Acids and Cardiovascular Events after Myocardial Infarction. N. Engl. J. Med..

[60] Jacobs M.L., Faizi H.A., Peruzzi J.A., Vlahovska P.M., Kamat N.P. (2021). EPA and DHA differentially modulate membrane elasticity in the presence of cholesterol. Biophys. J..

[61] Sherratt S.C.R., Libby P., Budoff M.J., Bhatt D.L., Mason R.P. (2023). Role of Omega-3 Fatty Acids in Cardiovascular Disease: The Debate Continues. Curr. Atheroscler. Rep..

[62] Bhatt D.L., Steg P.G., Miller M., Brinton E.A., Jacobson T.A., Ketchum S.B., Doyle R.T., Juliano R.A., Jiao L., Granowitz C. (2019). Cardiovascular Risk Reduction with Icosapent Ethyl for Hypertriglyceridemia. N. Engl. J. Med..

[63] Serini S., Calviello G. (2020). Omega-3 PUFA Responders and Non-Responders and the Prevention of Lipid Dysmetabolism and Related Diseases. Nutrients.

